# Supplementary material for: Efficacy of Oncolytic Herpes Simplex Virus T-VEC Combined with BET Inhibitors as an Innovative Therapy Approach for NUT Carcinoma
Source: Cancers (Basel). 2022 Jun 2;14(11):2761. doi: 10.3390/cancers14112761 (PMC9179288; doi:10.3390/cancers14112761)
Supplement: Supplementary file 1 [file cancers-14-02761-s001.zip › cancers-1744095-supplementary.pdf]

Supplementary Materials

Efficacy of Oncolytic Herpes Simplex Virus T-VEC Combined with BET Inhibitors as an Innovative Therapy Approach for NUT Carcinoma

Paul V. Ohnesorge, Susanne Berchtold, Julia Beil, Simone A. Haas, Irina Smirnow, Andrea Schenk, Christopher A. French, Nhi M. Luong, Yeying Huang, Birgit Fehrenbacher, Martin Schaller and Ulrich M. Lauer

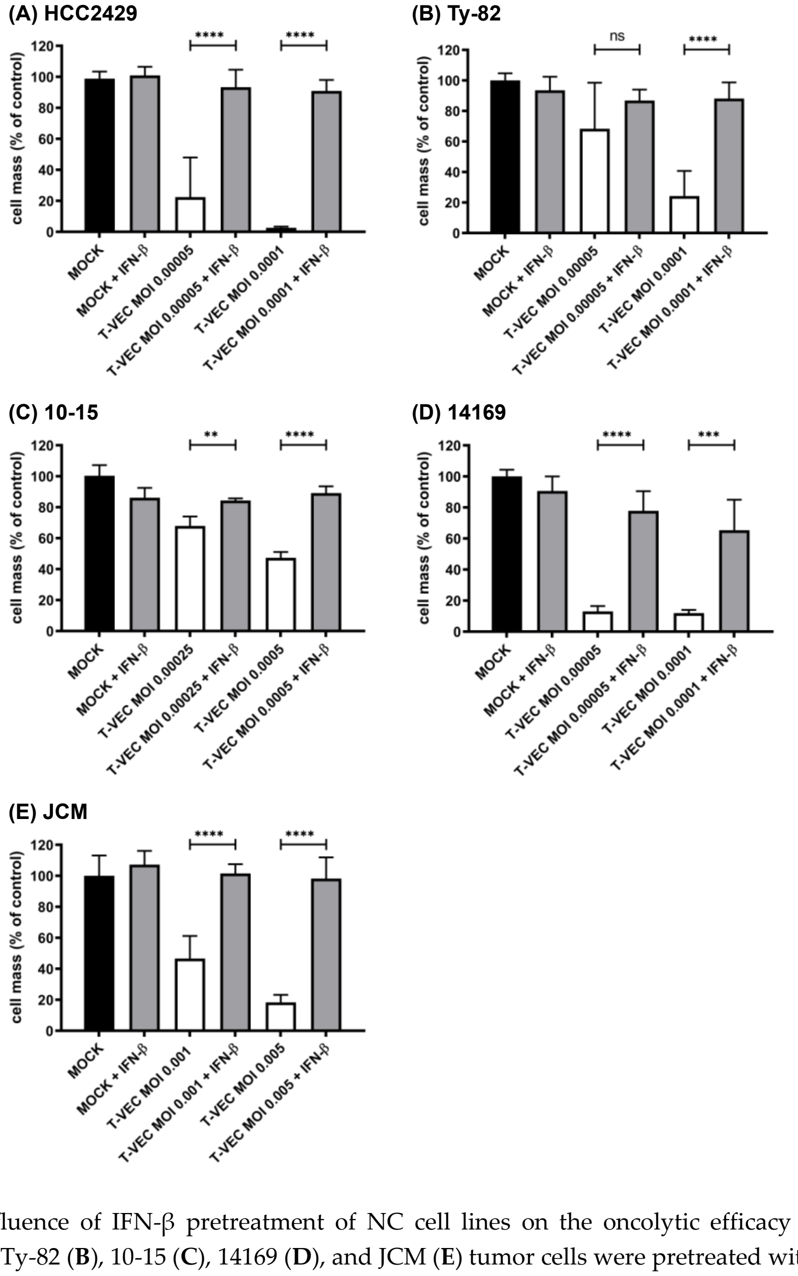

Figure S1. Influence of IFN-β pretreatment of NC cell lines on the oncolytic efficacy of T-VEC: HCC2429 (**A**), Ty-82 (**B**), 10-15 (**C**), 14169 (**D**), and JCM (**E**) tumor cells were pretreated with 2 ng/mL IFN-β for 16 h before infection with T-VEC at two different tumor cell line-adjusted multiplicities of infection (MOIs) or remained uninfected (MOCK + IFN-β). The remaining tumor cells were determined by SRB viability assay at 96 hours post infection (hpi). The anti-tumor effect of each treatment modality was calculated relative to untreated control (MOCK). The mean ± SD of at least two independent experiments performed in triplicates is shown. ** $p < 0.01$, *** $p < 0.001$, **** $p < 0.0001$, n.s. not significant.

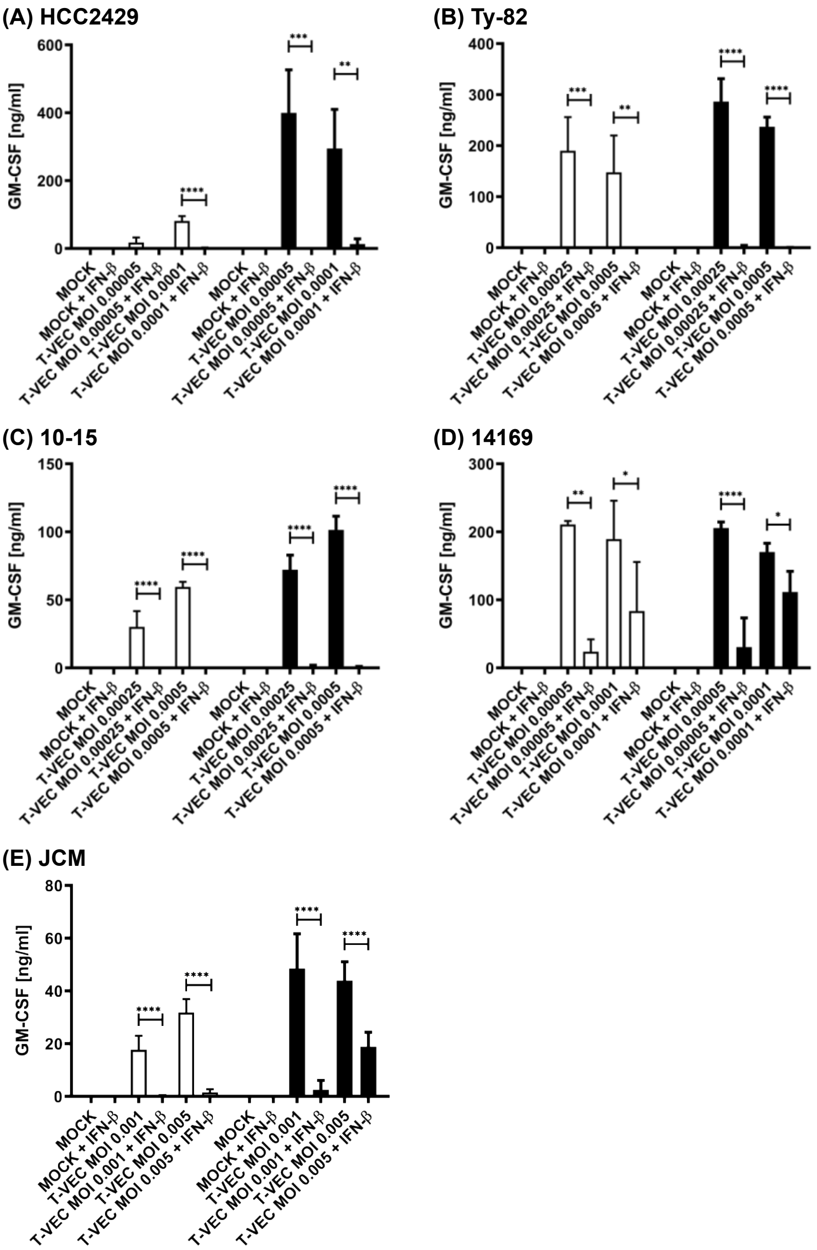

Figure S2. Influence of IFN-β pretreatment of NC cell lines on T-VEC-mediated GM-CSF expression: HCC2429 (**A**), Ty-82 (**B**), 10-15 (**C**), 14169 (**D**), and JCM (**E**) tumor cells were pretreated with 2 ng/mL IFN-β for 16 h before infection with T-VEC at two different tumor cell line-adjusted multiplicities of infection (MOIs) or remained uninfected (MOCK + IFN-β). At 72 hpi (white bars) and 96 hpi (black bars), supernatants were harvested and T-VEC-mediated expression of the marker protein GM-CSF was measured via ELISA. The mean ± SD of at least two independent experiments performed in triplicates is shown. * $p < 0.05$; ** $p < 0.01$, *** $p < 0.001$, **** $p < 0.0001$.

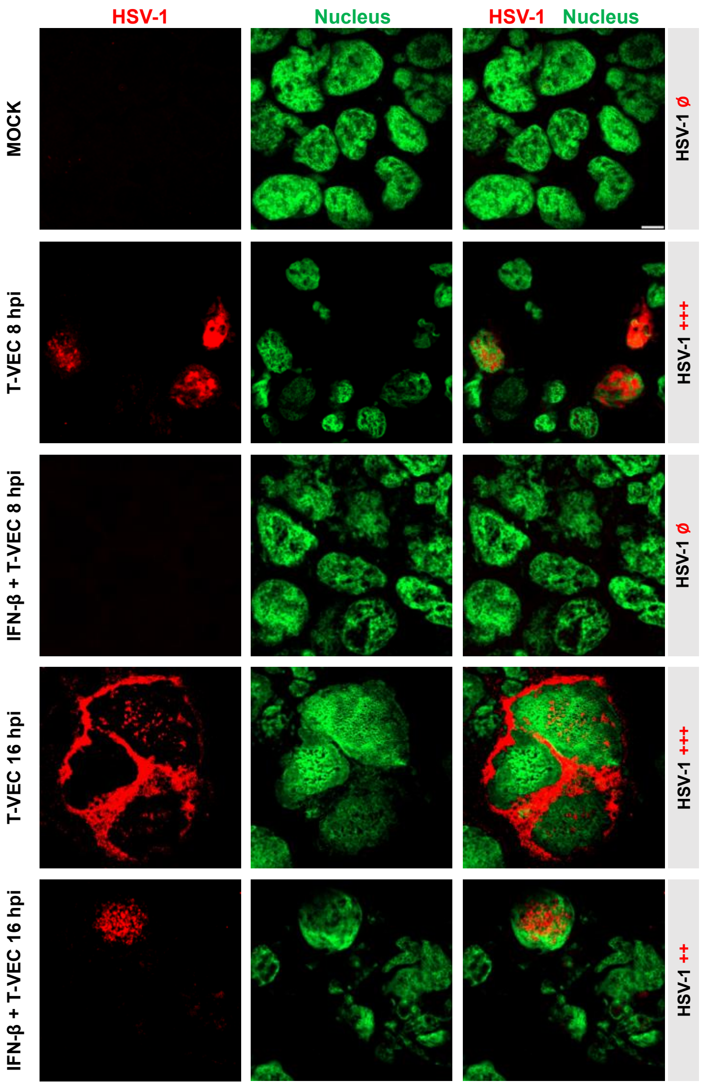

Figure S3. Influence of IFN-β pretreatment of NC cell line 143100 on the oncolytic efficacy of T-VEC: Supplementary data to Figure 3C, in which immunofluorescence images of 143100 cells pretreated with IFN-β and infected with T-VEC (MOI 0.1) for 8 or 16 hours are presented as merged images. Here, also, original single channel images of HSV-1 (red) and nucleus (green) stainings are depicted which were used to create the merged images. ++, moderate HSV-1 staining; +++, strong HSV-1 staining; Ø, no HSV-1 staining. Scale bar shows 5 µm.

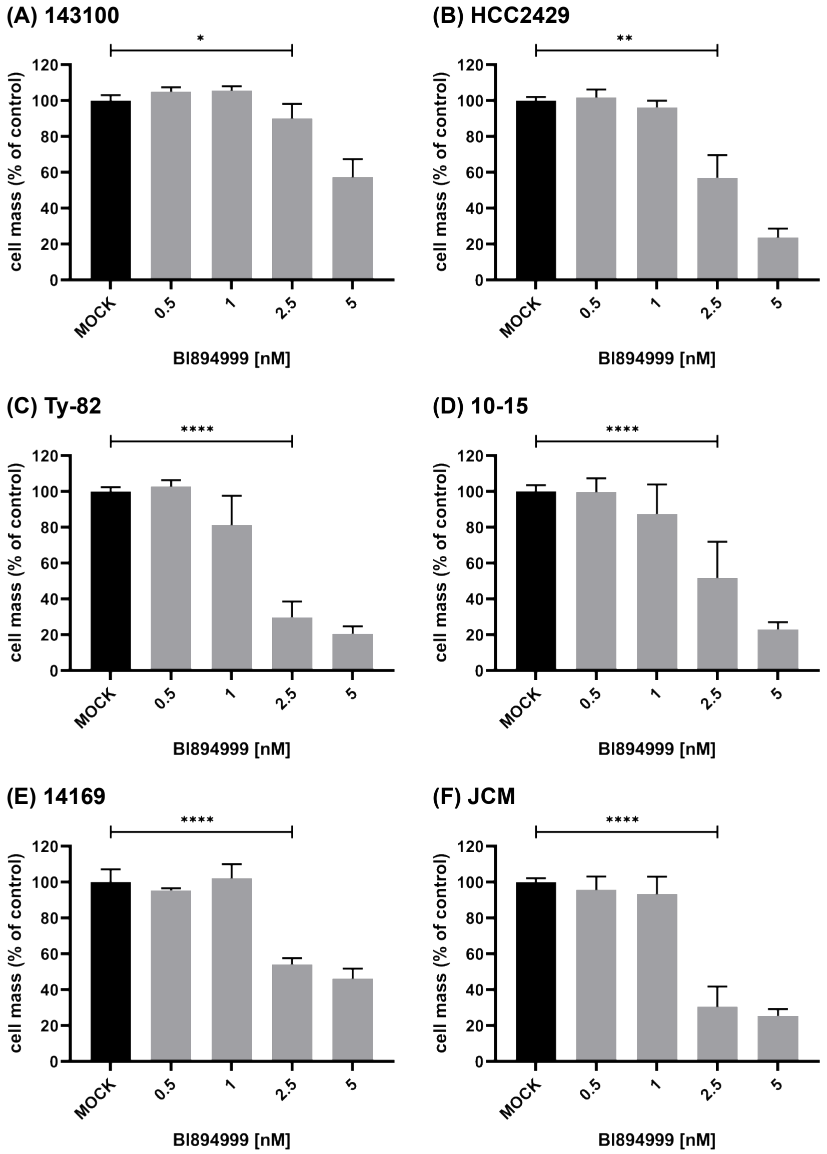

Figure S4. Viability of NC cell lines after monotherapeutic treatment with the BET inhibitor BI894999 (BI): 143100 (**A**), HCC2429 (**B**), Ty-82 (**C**), 10-15 (**D**), 14169 (**E**), and JCM (**F**) tumor cells were treated with BI at different concentrations ranging from 0.5 to 5 nM or remained untreated (MOCK). The remaining tumor cells were determined by SRB viability assay at 96 hours post treatment (hpt). The anti-tumor effect mediated by BI is calculated relative to MOCK control. The mean ± SD of at least two independent experiments performed in triplicates is shown. Displayed significances refer to the respective lowest BI concentration that causes a significant reduction in cell mass compared to MOCK. * $p < 0.05$; ** $p < 0.01$, **** $p < 0.0001$.

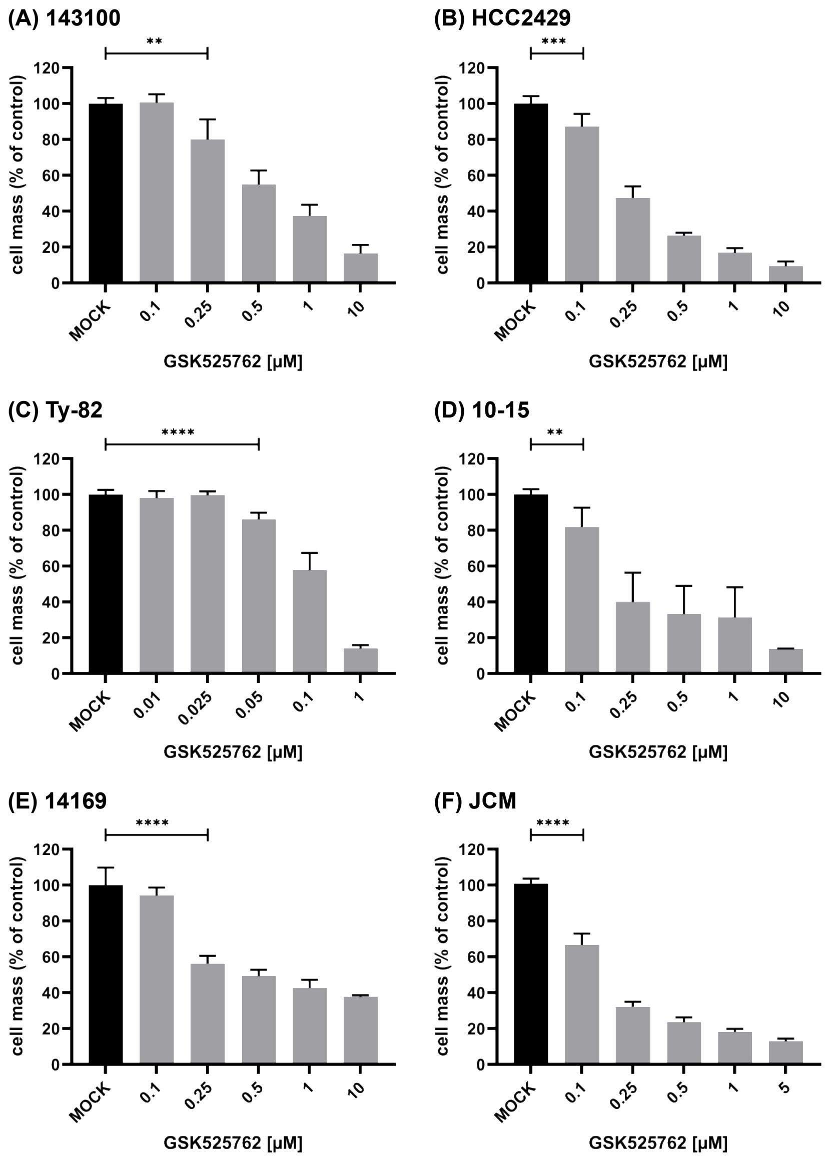

Figure S5. Viability of NC cell lines after monotherapeutic treatment with the BET inhibitor GSK525762 (GSK): 143100 (**A**), HCC2429 (**B**), Ty-82 (**C**), 10-15 (**D**), 14169 (**E**), and JCM (**F**) tumor cells were treated with GSK at different concentrations ranging from 0.1 to 10 µM or remained untreated (MOCK). The remaining tumor cells were determined by SRB viability assay at 96 hours post treatment (hpt). The anti-tumor effect mediated by GSK is calculated relative to MOCK control. The mean ± SD of at least two independent experiments performed in triplicates is shown. Displayed significances refer to the respective lowest GSK concentration that causes a significant reduction in cell mass compared to MOCK. ** $p < 0.01$, *** $p < 0.001$, **** $p < 0.0001$.

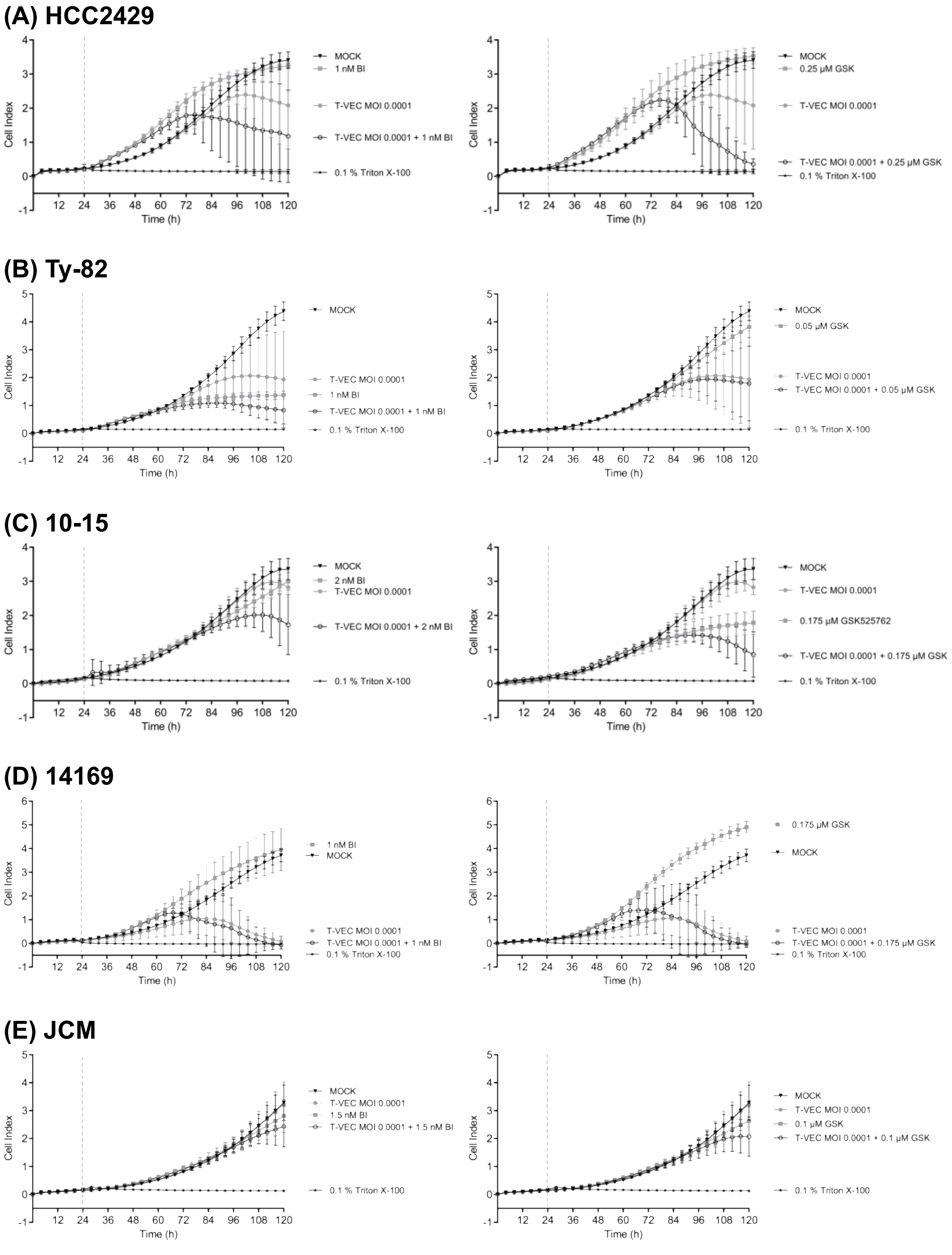

Figure S6. Real-time analysis of T-VEC-mediated oncolysis in NC tumor cell lines alone or after combinatorial treatment with the BET inhibitors BI894999 (BI) (left panels) or GSK525762 (GSK) (right panels): Twenty-four hours after seeding, HCC2429 (**A**), Ty-82 (**B**), 10-15 (**C**), 14169 (**D**), and JCM (**E**) tumor cells were infected with T-VEC (MOI 0.0001) alone or in combination with cell line-adjusted concentrations of BI (left panels) or GSK (right panels) or remained untreated (MOCK). Triton X-100 was added as a negative control inducing maximum lysis of tumor cells. Real-time cell proliferation was monitored using the xCELLigence® RTCA SP system. Measured electrode impedance is expressed as cell index. One representative of two independent experiments performed in triplicates is shown. Vertical dashed line indicates time point of T-VEC infection.
